# Incidental Changes in Urinary Lead Excretion Following Intra-Venous Ca-DTPA Administration in Patients Treated Primarily for Gadolinium Exposure

**DOI:** 10.3390/jox16040135

**Published:** 2026-07-23

**Authors:** Richard C. Semelka, Miguel Ramalho

**Affiliations:** 1 Richard Semelka Consulting, PLLC, 3901 Jones Ferry Road, Chapel Hill, NC 27516, USA; richardsemelka@gmail.com; 2 Department of Radiology, Hospital da Luz Lisboa, Avenida Lusíada 100, 1500-650 Lisbon, Portugal

**Keywords:** lead mobilization, urinary excretion, calcium–DTPA, gadolinium exposure, secondary analysis, age

## Abstract

Lead is a prevalent environmental pollutant and the heavy metal most frequently associated with toxic effects in humans. This study aimed to evaluate changes in urinary lead excretion following intravenous Ca-DTPA administration. We retrospectively included all patients treated at a medical clinic between January 2023 and March 2025 for suspected symptoms associated with gadolinium exposure who underwent intravenous Ca-DTPA chelation and had both pre- and post-chelation 24 h urine testing for 20 heavy metals. Urinary lead excretion was summarized using mean, standard deviation, median, and interquartile range, and non-parametric testing was applied because values were not normally distributed. A total of 63 participants were included, of whom 37 were female, with a mean age of 48.6 ± 12.5 years, ranging from 15 to 78 years. Median urinary lead excretion increased from 0.283 µg/24 h (IQR, 0.183–0.421) before treatment to 3.22 µg/24 h (IQR, 1.87–5.28) afterward (*p* < 0.001), corresponding to a median 11.8-fold increase. Participants aged ≥50 years had a higher median fold increase than younger participants (14.0 vs. 9.8; *p* = 0.003). All participants had detectable urinary lead levels, and intravenous Ca-DTPA administration was associated with a significant increase in urinary lead excretion. Older participants demonstrated greater lead mobilization and excretion than younger participants. These findings suggest that Ca-DTPA may reveal a mobilizable body burden of lead under the tested conditions, but they do not establish lead poisoning, reduction in total-body lead burden, symptom improvement, or long-term clinical benefit.

## 1. Introduction

Lead is a widespread environmental contaminant and the most reported heavy metal causing toxicity in humans [1,2,3]. Since 1997, the CDC’s Agency for Toxic Substances and Disease Registry (ATSDR) has listed lead as the second-highest health threat on its Substance Priority List due to its toxicity and likelihood of exposure [4]. People are mainly exposed to lead through ingestion and inhalation [2,5]. Human lead levels increased sharply in the 20th century because of the use of leaded gasoline, lead-based paints, and lead-containing water systems [2,6,7,8,9]. Even though regulations phased out lead-based paint in 1971 and banned leaded gasoline by 1988, recent studies still find lead in consumer products like baby formula and tampons [10,11,12]. As a result, low-level exposure continues. Besides postnatal exposure, lead can also be transferred from mother to child during pregnancy, which adds to early-life body burden [13,14]. Lead exposure is linked to higher cardiovascular risk and metal-induced autoimmunity [15]. There are three main ways to manage toxic metals like lead: avoidance, detoxification, and active removal. Most research has focused on avoidance and prevention, which are the main public health approaches [1]. In a recent review of lead toxicity, chelation therapy was only briefly mentioned, with the authors noting mixed results [1]. Blood lead levels are usually considered the main indicator of body burden.

**Figure 1:**
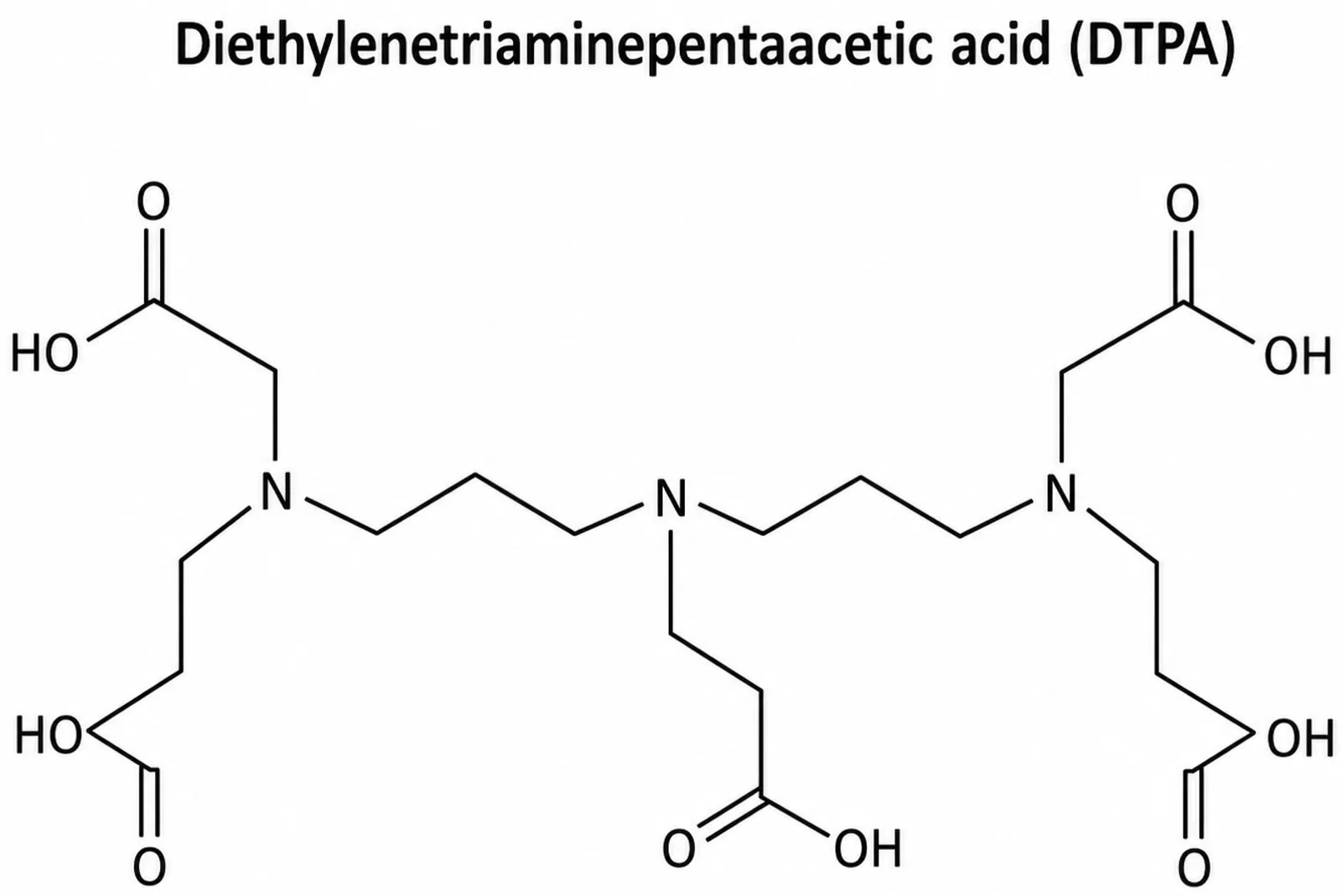
Chemical structure of diethylenetriaminepentaacetic acid (DTPA), a polyaminocarboxylate chelator containing three nitrogen and five carboxylate donor groups capable of coordinating metal ions.

Diethylenetriaminepentaacetic acid (DTPA) is a polyaminocarboxylate ligand with multiple metal-binding donor sites (Figure 1) [16]. Ca-DTPA is approved as a decorporation agent for internal contamination with plutonium, americium, or curium, but not for lead poisoning [17]. Because DTPA is not metal-specific, paired urine collections obtained during treatment for one metal may also demonstrate increased excretion of other metals.

In this study, Ca-DTPA was administered primarily to patients with suspected symptoms associated with gadolinium exposure (SAGE)/gadolinium deposition disease (GDD), rather than lead intoxication [18,19,20]. This secondary analysis aimed to characterize changes in 24 h urinary lead excretion after the first intravenous Ca-DTPA administration and to explore differences according to age. It was not designed to evaluate Ca-DTPA as a treatment for lead poisoning or to determine its effects on total-body lead burden, symptoms, or clinical outcomes. We also discuss relevant chelator properties, including metal-binding affinity, stability constants, and evidence of metal mobilization based on paired 24 h urine measurements.

## 2. Materials and Methods

Patients seen at a private medical clinic between January 2023 and March 2025, treated with intravenous IV-DTPA chelation for suspected symptoms associated with gadolinium exposure (SAGE)/gadolinium deposition disease (GDD), and who had pre- and post-chelation 24 h urine measurements for 20 heavy metals, were included in this study. Sixty-three subjects met these criteria and were included in this analysis. Sixty-one presented with a history of suspected gadolinium toxicity, one sought gadolinium removal without symptoms of toxicity, and one was evaluated for suspected lead toxicity.

The study population consisted of 37 women and 26 men (48.6 ± 12.5 years old), aged 15 to 78 years. Patients were categorized into two age groups: patients aged <50 and patients aged ≥50.

All participants had 24 h urine tests for 20 heavy metals at a commercial lab (Doctor’s Data, St. Charles, IL, USA) using dynamic reaction cell inductively coupled plasma–mass spectrometry (DRC-ICP-MS). Urine samples were collected in metal-free containers within 3 days before chelation and again 1 h after IV-Ca-DTPA administration. Urine lead concentration was measured in micrograms per 24 h. The detection limit for lead was 0.07 µg/g. Post-chelation urinary lead excretion was interpreted as a measure of mobilizable lead under the conditions tested and not as a direct measure of total body burden.

Chelation was performed with Ca-DTPA, with all patients receiving a standard dose of 5 mL (1 g) of Ca-DTPA. Only the first chelation session was analyzed to preserve consistency. Ca-DTPA was given as a manual bolus injection, followed by an intravenous saline infusion. Ca-DTPA acts by exchanging its calcium ion for lead (Pb^2+^). DTPA then binds the lead through multiple nitrogen and oxygen donor sites, forming a stable, water-soluble Pb–DTPA complex that is excreted in the urine [17].

All patients received IV steroids (63 mg or 125 mg) during chelation to reduce inflammation [16,17,18]. They also started a standard steroid (methylprednisolone) and antihistamine taper the day after chelation. Ca-DTPA and its metal chelates are primarily cleared by glomerular filtration. Corticosteroids may transiently affect renal hemodynamics, but there is no evidence that the administered corticosteroids or antihistamines meaningfully alter Ca-DTPA pharmacokinetics or urinary metal excretion.

This retrospective observational report was based on anonymized clinical data collected during routine care at a private medical clinic. No additional intervention or diagnostic procedure was performed for research purposes, and all data were de-identified before analysis. Under local regulatory requirements for retrospective analyses of anonymized routine clinical data, formal Institutional Review Board approval was not required. The study was conducted in accordance with the principles of the Declaration of Helsinki.

Statistical analysis was performed using paired and non-parametric methods. Because urinary lead excretion values showed a skewed distribution with several high post-chelation values, pre- and post-chelation urinary lead excretion was compared using the Wilcoxon signed-rank test, which is appropriate for paired non-normally distributed data. Between-group comparisons according to age category (<50 years vs. ≥50 years) were performed using the Mann–Whitney U test. Results are reported as mean and median with interquartile range, with emphasis on median values for descriptive interpretation. A *p*-value < 0.05 was considered statistically significant.

AI-assisted tools, specifically ChatGPT, GPT-5.5 Thinking model, OpenAI, were used to support the preparation and visual refinement of the graphical abstract and figures. All outputs were reviewed and approved by the authors, who remain fully responsible for the final content.

## 3. Results

**Figure 2:**
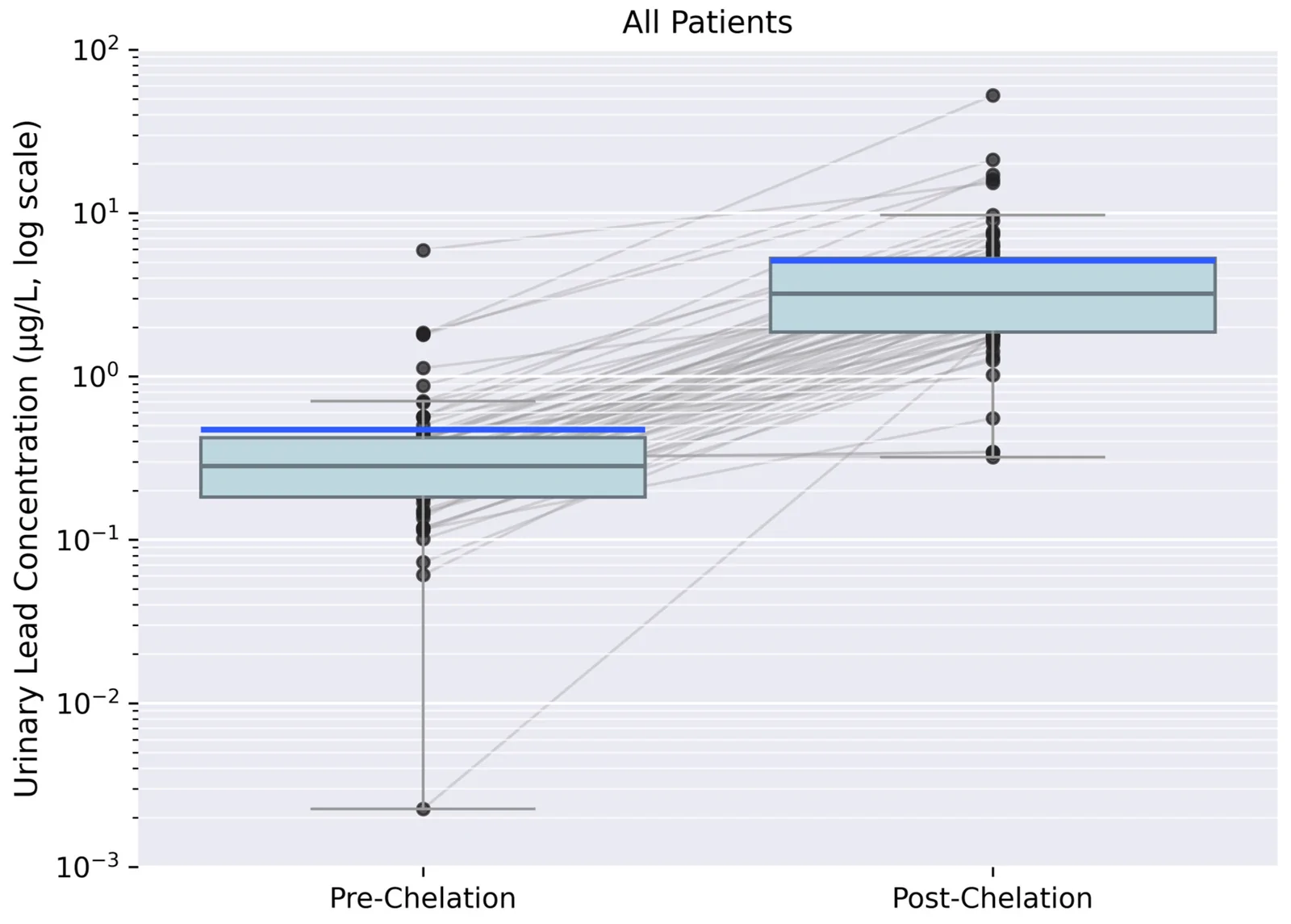
Within-participant change in 24-h urinary lead excretion before and after the first intravenous Ca-DTPA administration. Each black dot represents an individual 24-h urinary lead excretion value, and gray lines connect paired pre- and post-chelation values from the same participant. Boxes represent the interquartile range, horizontal lines within boxes indicate the median, whiskers show the data range according to standard boxplot conventions, and the blue horizontal line indicates the mean. The y-axis is shown on a logarithmic scale. Values are expressed as µg/24 h.

All 63 participants had detectable lead in their 24 h urine samples before and after chelation (Table 1). Pre-chelation values were much lower than post-chelation values (*p* < 0.05). Before chelation, urinary lead levels ranged from 0.02 to 1.8 µg/24 h (mean: 0.47 µg/24 h). After chelation, values ranged from 0.3 to 52.6 µg/24 h (mean: 5.1 µg/24 h) (Figure 2).

Median urinary lead excretion increased from 0.283 µg/24 h (IQR 0.183–0.421) before chelation to 3.22 µg/24 h (IQR 1.87–5.28) after chelation (Wilcoxon signed-rank test, *p* < 0.001). The median fold increase was 11.8-fold (IQR 8.7–16.7), with a maximum increase of 79-fold.

**Figure 3:**
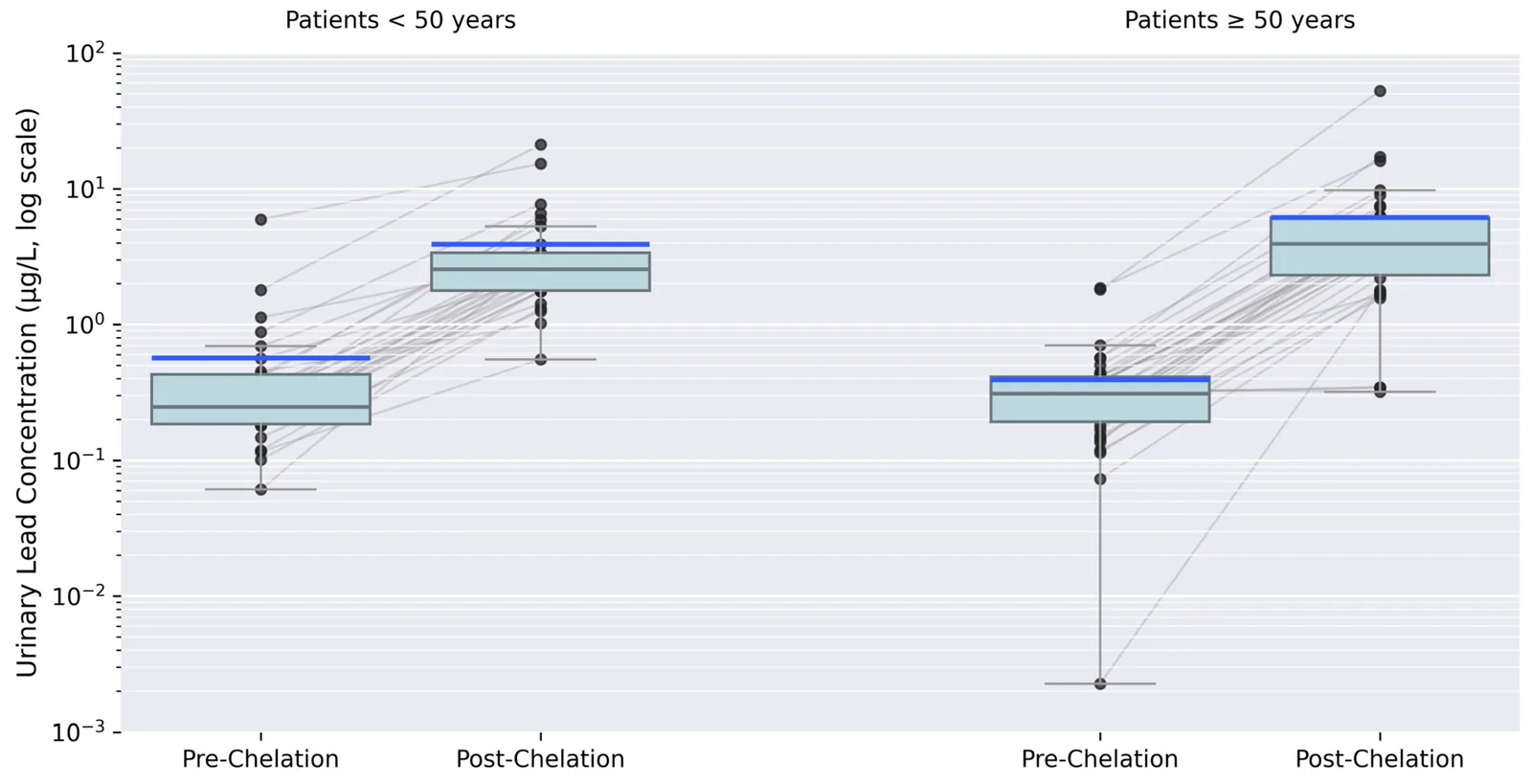
Within-participant change in 24-h urinary lead excretion before and after the first intravenous Ca-DTPA administration, stratified by age group. Results are shown separately for participants aged <50 years and ≥50 years. Each black dot represents an individual 24-h urinary lead excretion value, and gray lines connect paired pre- and post-chelation values from the same participant. Boxes represent the interquartile range, horizontal lines within boxes indicate the median, whiskers show the data range according to standard boxplot conventions, and the blue horizontal line indicates the mean. The y-axis is shown on a logarithmic scale. This age-stratified analysis is exploratory. Values are expressed as µg/24 h.

Participants aged ≥50 years showed greater post-chelation urinary lead excretion than younger participants. In the ≥50-year group (*n* = 34), median urinary lead excretion increased from 0.310 µg/24 h (IQR 0.192–0.412) before chelation to 3.93 µg/24 h (IQR 2.31–6.11) after IV Ca-DTPA administration. In comparison, participants aged <50 years (*n* = 29) increased from 0.248 µg/24 h (IQR 0.185–0.429) to 2.56 µg/24 h (IQR 1.78–3.37). The median fold increase was higher in the ≥50-year group than in the <50-year group (14.0-fold [IQR 11.2–19.0] vs. 9.8-fold [IQR 6.9–12.3]; Mann–Whitney U test, *p* = 0.003) (Figure 3). These findings suggest a greater mobilizable lead fraction in older participants, although this interpretation remains exploratory.

## 4. Discussion

Our findings showed that intravenous Ca-DTPA administered primarily for gadolinium-related conditions was followed by a reproducible increase in urinary lead excretion, with older individuals demonstrating higher burdens, likely reflecting cumulative exposure to lead from leaded paint, gasoline, and plumbing. Previous literature shows that approximately 95% of retained lead in adults resides in the skeleton, compared with 70% in children, while only about 1% circulates in the blood [1]. Thus, skeletal stores likely act as a long-term reservoir, slowly releasing lead even after exogenous exposure is reduced [21]. The relationship between blood levels, urinary excretion, and total body burden remains incompletely defined. Although blood lead concentration remains the clinical standard, we believe it is likely a poor indicator of actual body lead content, as it reflects only a small circulating fraction. In contrast, post-chelation 24 h urine collections, particularly when high-affinity agents such as DTPA or edetate calcium disodium (CaNa_2_EDTA) are used, may provide complementary information regarding mobilizable lead fractions, although their relationship to total body lead burden remains incompletely defined. Blood lead levels vary with timing, circadian factors, and chelation cycles, whereas paired pre- and post-chelation 24 h urine testing more precisely reflects mobilizable lead burden.

Two characteristics are central to evaluating chelator suitability: (i) log stability constant, which reflects metal-ligand binding affinity; and (ii) demonstrated success in urinary metal excretion, assessed by pre- and post-chelation 24 h urine levels.

EDTA is an FDA-approved, established first-line therapy for lead intoxication and has decades of clinical use. Its log K for lead (~18.0) is only slightly lower than that of DTPA (~18.8) [22,23,24,25], supporting its long-recognized success in mobilizing both soft-tissue and skeletal lead stores [26]. Previous studies have shown that EDTA can substantially reduce blood lead concentrations (up to 61%) and increase urinary excretion [27]. However, consistent reductions in cardiovascular events have not been demonstrated.

Preclinical data demonstrate that DTPA can mobilize lead from various tissues, including the brain, and facilitate its excretion [28]. Nonetheless, clinical evidence in humans is very limited. A previous study by Llobet et al. [29] showed that both EDTA and DTPA effectively enhanced lead elimination in mice, with DTPA producing a greater reduction. In our study, the first post-DTPA infusion produced a mean 26-fold increase in urinary lead (range up to 79-fold), with greater increases in patients older than 50 years (mean 38.7-fold) than in younger individuals (11.3-fold). These values are comparable to findings with EDTA. Alam et al. [30] reported a 35.8-fold increase (range 16.7–79-fold) in urinary lead after the initial EDTA infusion in patients aged 50 years or older. Potential biological mechanisms for this age-related rise may entail greater cumulative exposure over time and changes in bone metabolism that influence lead release. For instance, aging may be associated with increased bone resorption, which could release more lead from bone stores into the bloodstream. Furthermore, age-related declines in kidney function may reduce the body’s ability to clear lead, thereby increasing overall lead retention. Investigating these mechanisms provides a platform for further investigation aimed at understanding how lead retention and excretion processes evolve in older adults.

Provoked urine measurements are not recommended as diagnostic tests for metal poisoning because chelator administration changes urinary excretion and validated thresholds for interpreting the resulting values are lacking [31].

The study also does not demonstrate clinical benefit from enhanced lead excretion.

Ca-DTPA and EDTA should not be treated as interchangeable clinical therapies. EDTA is an established, approved treatment for acute and chronic lead poisoning, whereas Ca-DTPA is approved for decorporation of selected transuranium elements and lacks adequate human evidence for treating lead intoxication. Although both are chelators with high metal-binding capacity, in vitro affinity does not establish clinical superiority, and the current study contains no direct comparison with EDTA. Table 2 summarizes the distinctions relevant to the interpretation of this cohort.

Safety is especially relevant when Ca-DTPA is administered repeatedly. The prescribing information warns that Ca-DTPA can deplete endogenous Zn, Mg, Cu, and Mn; the magnitude increases with dose, divided dosing, and treatment duration. Significant depletion has not been observed over nine years of our clinical experience. This is likely related to the 3–4-week intervals between chelation sessions, which allow sufficient time for metabolic re-equilibration.

Like gadolinium, most lead is stored in bone [1]. Repeated chelation may alter exchangeable lead pools over time, although the kinetics of redistribution between tissue compartments remain incompletely understood. In our experience, progressive reduction in post-chelation urinary lead is commonly seen only after five or more treatment cycles, as significant reductions in bone lead content are a prolonged process. These findings are descriptive and hypothesis-generating and require validation in controlled studies.

The consistency of within-subject increases in urinary lead excretion across all 63 participants strengthens the internal validity of the observed association, although the clinical significance of this finding remains uncertain. Therefore, additional investigation is needed to investigate whether there is a correlation between post-chelation urine levels and actual clinical outcomes.

This study has several limitations. It was retrospective and not designed around lead exposure or lead-related outcomes. There was no untreated control group; blood lead and bone lead were not measured; the timing and completeness of 24 h collections could not be independently verified; concomitant medications were administered; and one participant had a distinct lead-related clinical indication. The use of medians and rank-based methods reduces, but does not eliminate, the influence of this single outlier and does not correct the broader limitations.

## 5. Conclusions

Ca-DTPA was associated with increased urinary lead excretion in this cohort; however, these findings are descriptive and hypothesis-generating. Older participants showed a greater fold increase. These findings support incidental lead mobilization and urinary excretion after Ca-DTPA and generate a hypothesis about age-related differences in chelator-accessible lead. They do not establish lead poisoning, diagnostic utility of provoked urine testing, reduction in total-body lead stores, superiority to EDTA, or clinical benefit.

## Figures and Tables

**Table 1:** Participant age and urinary lead excretion before and after chelation.

Variable	Summary Statistic	Value
Participants, *n*		62
Age, years	Mean ± SD	48.6 ± 12.7
Age, years	Median (IQR)	50.5 (40.0–56.8)
Pre-chelation urinary lead, µg/24 h	Median (IQR)	0.276 (0.182–0.412)
Post-chelation urinary lead, µg/24 h	Median (IQR)	3.208 (1.834–5.237)
Absolute change, µg/24 h	Median (IQR)	2.965 (1.671–4.863)
Fold change	Median (IQR)	11.66 (8.70–16.07)
Paired comparison, *p*-value	Wilcoxon signed-rank test	<0.001

Data are presented as mean ± standard deviation (SD) or median (interquartile range, IQR), as indicated. The paired pre- and post-chelation concentrations were compared using the Wilcoxon signed-rank test. Units should be verified against the analytical laboratory report.

**Table 2:** Comparison of edetate calcium disodium and calcium-DTPA in relation to lead chelation.

Feature	Edetate Calcium Disodium (CaNa_2_EDTA)	Calcium–DTPA (Ca-DTPA)
**Ligand architecture and metal binding**	EDTA-based aminopolycarboxylate with high affinity for Pb(II) and clinically established lead-chelating activity.	DTPA-based aminopolycarboxylate with additional donor atoms. It can bind Pb(II), but its clinical efficacy for lead poisoning has not been established.
**Approved indication**	Reduction in blood lead concentrations and body stores in acute and chronic lead poisoning, including lead encephalopathy.	Enhancement of elimination after known or suspected internal contamination with plutonium, americium, or curium.
**Evidence in lead poisoning**	Established clinical use; treatment is guided by blood lead concentration, symptoms, and clinical status.	Limited to preclinical studies and observational urinary-excretion data; no validated therapeutic role in lead poisoning.
**Administration in approved use**	Intravenous or intramuscular.	Intravenous or inhaled. The labeled adult initial dose is 1 g.
**Major safety considerations**	Dose-related nephrotoxicity; renal function and urine output should be monitored. Zinc depletion may occur.	May deplete zinc, magnesium, and manganese, particularly with repeated or divided dosing. Monitoring may include renal function, electrolytes, urinalysis, blood counts, and trace metals.
**Interpretation in the present study**	Not administered; no direct comparison of efficacy or safety can be made.	Study exposure. Increased urinary lead indicates mobilization under the sampling conditions, not a proven reduction in total-body lead burden or clinical benefit.

Abbreviations: Ca-DTPA, calcium diethylenetriaminepentaacetate; CaNa_2_EDTA, edetate calcium disodium; EDTA, ethylenediaminetetraacetic acid; Pb(II), divalent lead.

## Data Availability

The original contributions presented in this study are included in the article/Supplementary Material. Further inquiries can be directed to the corresponding author.

## Supplementary Materials

The following supporting information can be downloaded at: https://www.mdpi.com/article/10.3390/jox16040135/s1, Table S1: Individual participant data.
