# Supplementary material for: Incidental Changes in Urinary Lead Excretion Following Intra-Venous Ca-DTPA Administration in Patients Treated Primarily for Gadolinium Exposure
Source: J Xenobiot. 2026 Jul 23;16(4):135. doi: 10.3390/jox16040135 (PMC13514330; doi:10.3390/jox16040135)
Supplement: Supplementary file 1 [file jox-16-00135-s001.zip › jox-4404465-supplementary.pdf]

Supplementary Materials: Incidental Changes in Urinary Lead Excretion Following Intra-Venous Ca-DTPA Administration in Patients Treated Primarily for Gadolinium Exposure

Richard C. Semelka and Miguel Ramalho

Table S1. Individual participant data.

Participant	Age	Pre-Chelation	Post-Chelation	Absolute Change	Fold Change
1	15	0.692	3.899	3.207	5.64
2	21	0.101	1.309	1.208	12.96
3	26	0.185	2.504	2.319	13.54
4	26	0.451	1.020	0.569	2.26
5	26	0.334	2.868	2.533	8.58
6	29	0.118	1.776	1.658	15.05
7	32	0.306	2.119	1.812	6.92
8	32	0.229	2.560	2.332	11.20
9	34	0.561	2.174	1.613	3.88
10	35	0.116	0.554	0.438	4.77
11	35	0.248	6.556	6.308	26.39
12	36	0.147	1.254	1.107	8.52
13	40	0.198	1.936	1.738	9.76
14	40	0.247	1.958	1.711	7.92
15	40	0.180	1.760	1.580	9.78
16	40	0.189	1.756	1.566	9.27
17	40	0.269	1.421	1.153	5.29
18	43	0.232	2.661	2.429	11.47
19	44	0.879	7.699	6.820	8.76
20	45	0.429	5.284	4.855	12.31
21	46	1.127	2.847	1.720	2.53
22	46	0.061	3.198	3.137	52.47
23	46	0.181	3.371	3.190	18.61
24	46	0.283	2.794	2.511	9.88
25	46	0.410	5.901	5.491	14.40
26	47	0.314	3.276	2.962	10.43
27	49	5.932	15.303	9.371	2.58
28	49	1.797	21.174	19.377	11.79
29	49	0.196	2.169	1.974	11.09
30	50	0.152	1.755	1.603	11.53
31	50	0.119	1.562	1.443	13.17
32	51	0.114	2.196	2.082	19.27
33	51	0.002	1.799	1.797	796.48
34	52	0.433	1.645	1.211	3.79
35	52	0.169	5.282	5.113	31.35
36	52	0.144	2.647	2.503	18.39
37	53	0.375	4.180	3.805	11.15
38	53	0.381	3.705	3.324	9.72
39	54	0.225	3.277	3.052	14.55
40	54	0.568	8.979	8.411	15.81
41	54	0.377	4.283	3.905	11.35
42	54	0.236	5.102	4.866	21.62
43	54	0.181	3.124	2.943	17.25
44	55	0.308	3.379	3.071	10.95
45	56	0.234	3.779	3.545	16.16
46	56	1.853	16.086	14.232	8.68
47	57	0.411	5.081	4.670	12.37
48	57	0.247	4.736	4.489	19.19
49	57	0.568	17.154	16.586	30.19

Participant	Age	Pre-Chelation	Post-Chelation	Absolute Change	Fold Change
50	58	0.252	3.825	3.573	15.17
51	58	0.249	3.217	2.968	12.94
52	59	0.136	7.314	7.178	53.66
53	61	0.704	9.747	9.043	13.84
54	61	0.383	4.034	3.650	10.52
55	64	0.311	0.346	0.034	1.11
56	64	0.333	0.320	-0.013	0.96
57	65	0.503	6.267	5.764	12.47
58	65	0.315	0.343	0.028	1.09
59	67	0.073	1.681	1.608	23.07
60	68	0.413	7.513	7.100	18.20
61	69	0.445	6.300	5.855	14.15
62	78	0.290	5.655	5.365	19.49

Pre-chelation, post-chelation, and absolute-change values are shown to three decimal places; fold change is shown to two decimal places. The source dataset supplied for this table contained 62 participants.
